# Field-Based Optimal Placement of Antennas for Body-Worn Wireless Sensors

**DOI:** 10.3390/s16050713

**Published:** 2016-05-17

**Authors:** Łukasz Januszkiewicz, Paolo Di Barba, Sławomir Hausman

**Affiliations:** 1 Institute of Electronics, Lodz University of Technology, ul. Wólczańska 211/215, 90-924 Łódź, Poland; lukasz.januszkiewicz@p.lodz.pl; 2 Department of Electrical, Computer and Biomedical Engineering, University of Pavia, via Ferrata 5, 27100 Pavia, Italy; paolo.dibarba@unipv.it

**Keywords:** body area networks, wireless sensor network, computer optimization algorithms, computational electromagnetics

## Abstract

We investigate a case of automated energy-budget-aware optimization of the physical position of nodes (sensors) in a Wireless Body Area Network (WBAN). This problem has not been presented in the literature yet, as opposed to antenna and routing optimization, which are relatively well-addressed. In our research, which was inspired by a safety-critical application for firefighters, the sensor network consists of three nodes located on the human body. The nodes communicate over a radio link operating in the 2.4 GHz or 5.8 GHz ISM frequency band. Two sensors have a fixed location: one on the head (earlobe pulse oximetry) and one on the arm (with accelerometers, temperature and humidity sensors, and a GPS receiver), while the position of the third sensor can be adjusted within a predefined region on the wearer’s chest. The path loss between each node pair strongly depends on the location of the nodes and is difficult to predict without performing a full-wave electromagnetic simulation. Our optimization scheme employs evolutionary computing. The novelty of our approach lies not only in the formulation of the problem but also in linking a fully automated optimization procedure with an electromagnetic simulator and a simplified human body model. This combination turns out to be a computationally effective solution, which, depending on the initial placement, has a potential to improve performance of our example sensor network setup by up to about 20 dB with respect to the path loss between selected nodes.

## 1. Introduction

Technological development, especially in the field of sensor technology, highly integrated electronics and telecommunications, allows for the introduction of new solutions in health monitoring of medical patients as well as monitoring of sportsmen, people working in hazardous environments or exposed to hazards at work, and other safety critical contexts [1]. Early solutions used wired sensors, involving wires and connectors, which adversely affected the user’s comfort. A more recent approach is to use body-worn wireless sensor networks, known as Wireless Body Area Networks (WBAN) [2,3]. Due to the development of the integrated circuit technology it is possible to manufacture ever more complex and yet miniature electronic modules (including sensors), small enough to be integrated into clothing. A good example are Flora sensors made by the company Adafruit [4]. Their diameter is less than 45 mm and the weight is 4.4 g. Such clothing, fitted with miniature sensors, electronic devices and computing power of microcontrollers is known as smart clothing or e-textiles [5]. The functions performed by smart clothing may be diverse, including a mobile, remote measurement of human physiological parameters, vital signs monitoring, monitoring of physical activity or automatic detection of risks. A comprehensive introduction to WBAN and relevant design issues are provided in [1].

**Figure 1:**
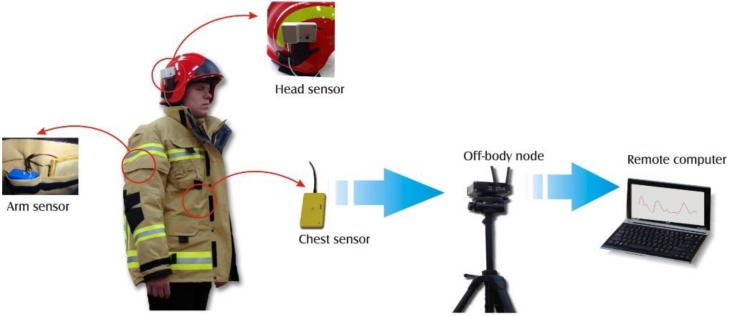
Structure of the wireless body-worn sensor system.

In this paper we discuss optimization of a WBAN system which uses wireless communication technology to monitor physiological and environmental parameters of firefighters and their exposure to hazardous environmental conditions. The system concept is presented in Figure 1. It consists of a head sensor, an arm sensor, and a chest sensor. The head sensor location is fixed in the helmet because it measures blood oxygen saturation (pulse oximetry) on the earlobe, which is one of typical locations used for this type of sensor [6]. The location of the arm node/sensors (accelerometers and a GPS receiver, temperature and humidity sensors) is fixed and determined by our experiments with a firefighter’s comfort for a particular design of protective clothing in mind. The chest node is both an on-body sensor and an off-body node which is communicating with on-body components and with the remote monitoring system. The chest node measures the humidity, temperature, and respiratory activity of firefighters and therefore is located on the chest, in a position ensuring a low value of the path loss to the other two on-body sensors to achieve low required RF transmission power and consequently low power consumption leading to an increased network lifetime or less demanding energy harvesting. The objective of our optimization procedure is to minimize the path loss between the chest sensor and the other two sensors: it is achieved by changing the location of the chest sensor within a predefined region, the boundary of which represents a problem constraint. In that respect our optimization problem is significantly different from those where the number of WBAN nodes (sensors and sinks) is greater and network topology as well as routing optimization should be considered, e.g., [7,8,9]. We do not assume any empirical-statistical radio wave propagation model as in [9]. We use more accurate computational electromagnetics methods as explained in Section 2.1.

**Figure 2:**
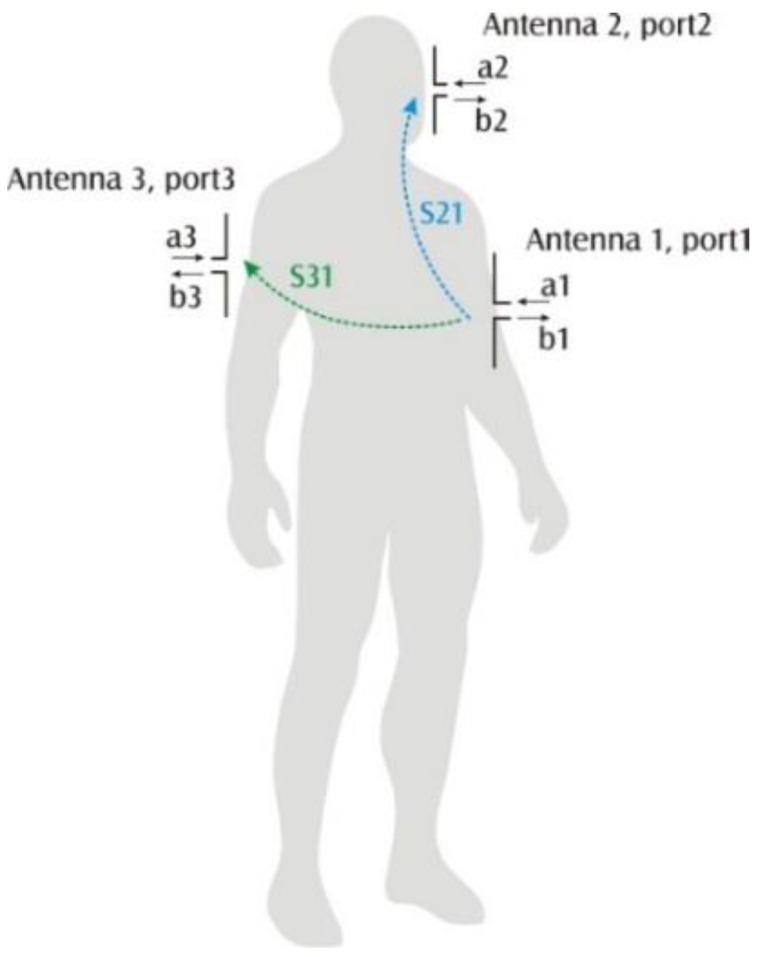
Scattering matrix parameters definition: *S*_21_ = *b*_2_/*a*_1_|*_a_*_2 = 0 and *a*3 = 0_ *S*_31_ = *b*_3_/*a*_1_|*_a_*_2 = 0 and *a*3 = 0_.

The path loss between the chest node and the other two nodes is defined by *S*_21_ and *S*_31_ which are parameters of the scattering matrix, widely used in the microwave technology. The parameters are defined as:

*S*_21_ = *b*_2_/*a*_1_ while *a*_2_ = 0 and *a*_3_ = 0 *S*_31_ = *b*_3_/*a*_1_ while *a*_2_ = 0 and *a*_3_ = 0 (1)

where: *a_i_*—input signal on port *i* and *b_j_*—output signal on port *j*, which are explained in Figure 2. We assume that the ports for which the scattering parameters are defined are the feeding points of the antennas. In our investigation, half wave dipole antennas in a vertical position were considered.

## 2. Methods

### 2.1. The Numerical Human Body Model

An analysis of WBANs and sensor network operating in the proximity of a human body is often carried out with the use of full-wave electromagnetic simulation software which also implements a numerical model of the human body. The Finite Difference Time Domain (FDTD) method is widely used for this type of studies [10]. In our research we use the XFdtd^®^ FDTD implementation from REMCOM, Inc. (State College, PA, USA) [11]. This package supports a scripting language which allows the user to execute customized functions, including real-time data exchange with external software.

A number of numerical human body models have been developed for the purpose of calculating the amount of electromagnetic power absorbed by the human body [12,13] (*i.e.*, Specific Absorption Rate) which can be also used for WBAN design. Currently, complex heterogeneous models which represent the inner structure of the human body reflecting electric properties of various tissue types are widely used. Those models are available in many contemporary electromagnetic CAD packages. A limitation of the multi-tissue, heterogeneous numerical models is the lack of their physical phantom equivalents because representations of the various internal organs would be very difficult to manufacture. Such models also require a lot of computer resources as the spatial discretization has to be dense to represent structural details of inner organs.

Experiments have been reported with the use of Specific Anthropomorphic Mannequin (SAM) which is a homogenous human body phantom [14,15]. The inner structure of the body is represented with homogenous tissue stimulant liquid with electromagnetic properties similar to the average value for the human body. Such models have a shape, proportions and general dimensions similar to an average human body. Those phantoms are expensive and difficult in use in the laboratory practice because they have to be filled with a liquid of precisely controlled electric properties which tend to vary with time and temperature. Simplified human body models have been also proposed for experimental studies e.g., [16] and for numerical simulation of WBANs [17,18]. They simplify a human body shape with cylinders filled with a homogenous tissue stimulant liquid but still are difficult to be re-created and require a careful control of dielectric properties of the liquid. This motivated us to propose an even more reduced complexity model which makes it very easy both for numerical implementation and manufacturing.

**Figure 3:**
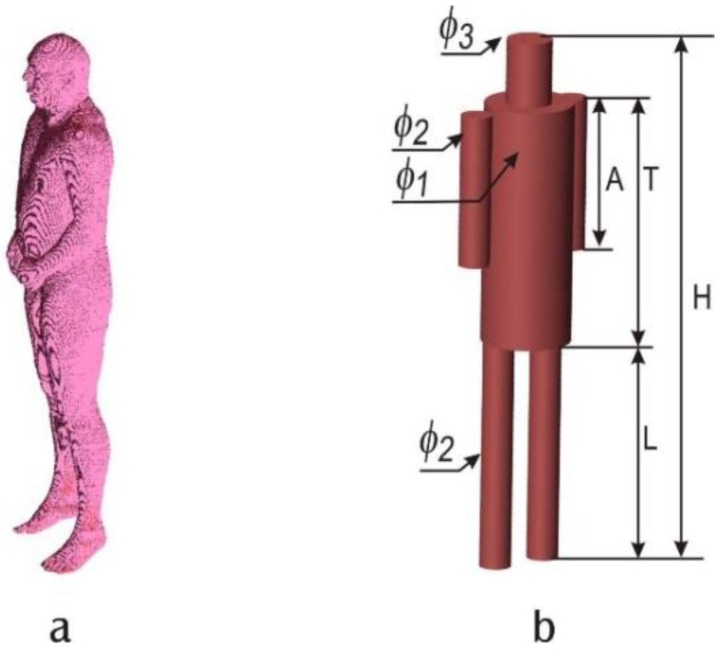
Human body model: (**a**) NMR Hershey; (**b**) simplified 6 cylinder model: H = 1800 mm, A = 500 mm, L = 790 mm, T = 836 mm, φ_1_ = 110 mm, φ_2_ = 160 mm, φ_3_ = 315 mm.

The simplified cylindrical model was also simulated with XFdtd^®^. Its geometry was optimized to approximate the proportion of the heterogeneous, anthropomorphic NMR Hershey model with six cylinders [19,20]. In Figure 3a, a reference NMR Hershey model is presented. The six cylinder model is presented in Figure 3b. Both the reference and the simplified models have the same height, H = 1800 mm. Other dimensions of the simplified model were fixed to fit the geometry of the reference model best, and to preserve the diameters of standard PVC pipes. The arm length is equal to A = 500 mm and the length of the legs is L = 790 mm, while the torso length is equal to T = 836 mm. The simplified model was designed in the way which makes it easy to manufacture. In order to be able to use the standard pipes available on the market, the following diameters of the cylinders were used in the model (see Figure 3): φ_1_ = 110 mm, φ_2_ = 160 mm, φ_3_ = 315 mm. The pipe wall thickness varies from 3 to 6 mm depending on the pipe diameter. All pipes were filled with liquid (a water solution of sucrose and kitchen salt) of electrical properties similar to the human tissue. The electric constant and the conductivity of the liquid were examined with Di-Line measurement unit from the IndexSar (Newdigate, UK). It employs a slot transmission line which is filled with the liquid under test. Material parameters are then identified from network parameters measured with a vector network analyzer. The electric constant was equal to ε ≈ 54 and the conductivity σ ≈ 1.5 S/m. These values are close to the average electric parameters of human tissues (ε = 52, σ= 1.8 S/m [20]). Values of ε = 54 and σ = 1.5 S/m were also used in the simulations presented later in the paper.

A cylindrical human body model was used for further investigation to achieve independence of rotation angle phi and distance *d* of the antenna that was moved across the chest. For an irregular body shape that would be difficult (there is also a difficulty in defining and calculating distance *d*). For the simplified cylindrical phantom it is straightforward.

**Figure 4:**
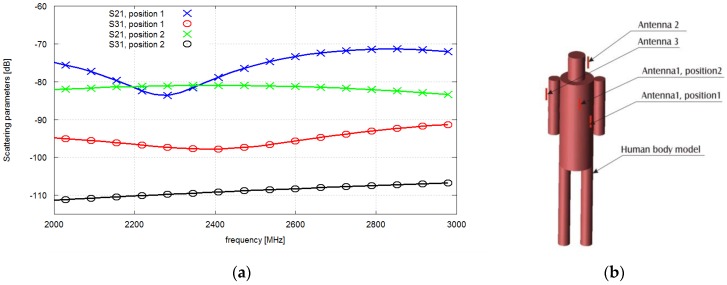
Scattering matrix parameter *vs.* frequency (**a**) and position of the chest node (**b**).

The motivation for the following work is the fact that the values of *S*_21_ and *S*_31_ strongly depend on the chest node antenna position, not just the antenna distance. This is evident in the example of simulation results obtained for two different locations of the chest antenna, which is presented in Figure 4.

The antennas in this case were tuned to 2.4 GHz. The position 1 of the chest node corresponds to the initial location of the pocket in which the node was placed. In this case *S*_21_ = −79 dB and *S*_31_ = −98 dB. The position 2 of antenna No. 1 corresponds to the closest possible location of this antenna to the antenna No. 3 within the feasibility region (a possible location of the pocket). Even though the distance is smaller, the attenuation of the radio wave is higher due to propagation effects in the proximity of the body. For this case *S*_21_ = −81 dB and *S*_31_ = −110 dB. This is motivates us to carry out a thorough study of antenna placement with optimization algorithm and it makes the distance based approach inapplicable. The path loss also depends significantly on the surrounding multipath environment, but for clarity we neglect this issue in the paper and concentrate on a free-space case. A discussion of an indoor multipath propagation scenario can be found in [21].

### 2.2. Statement of the Optimal Design Problem

The optimal design problem can be posed as follows:

*Having fixed the position of both a head sensor (antenna 2) and an arm sensor (antenna 3), find the position of the chest sensor (antenna 1) minimizing both S*_21_ *and S*_31_ *scattering parameters in the frequency band B between f_L_ and f_H_ (for 2.4 GHz ISM band we assumed that f_L_ = 2.4 GHz and f_H_ = 2.5 GHz while for 5GHz ISM band f_L_ = 5.7 GHz and f_H_ = 5.9 GHz)*.

**Figure 5:**
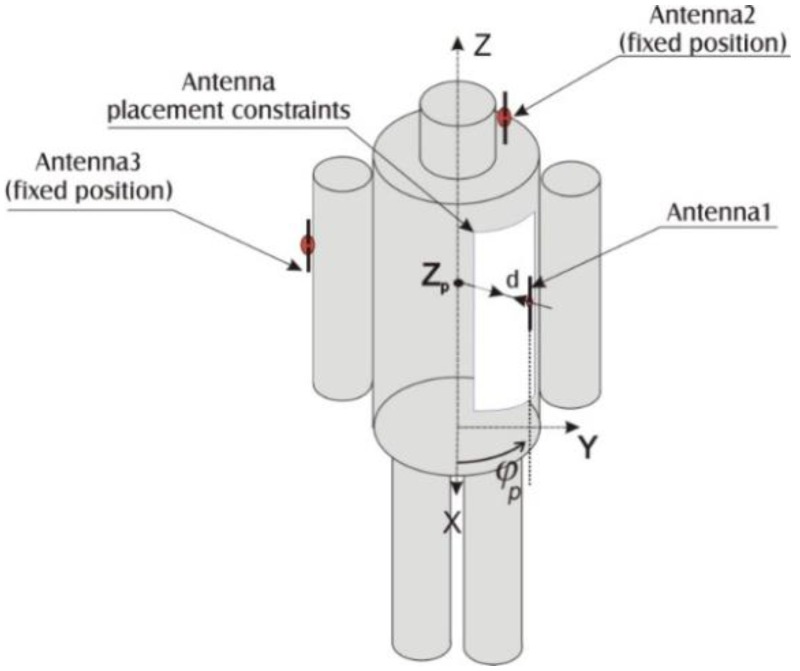
Six-cylinder model and feasible region (chest node region).

The position of antenna 1 is defined by distance *d*, height *z_p_* and azimuthal angle ϕ*_p_*. Therefore, for a given frequency *f*, the scattering parameters depend on the triplet of design variables (*d*, *z_p_*, ϕ*_p_*); the formal statement follows:

*Starting from a guess solution* *x*_0_, *find*

(2) $$\underset{x\in \Omega }{\inf}\;\underset{f\in B}{\sup}\;\left[S_{21}\left(x,f\right),S_{31}\left(x,f\right)\right]\;,\;x=\left(d,z_{p},\varphi_{p}\right)\;,\;\Omega \subset {ℜ}^{3}\;,\;B=\left[f_{L},f_{H}\right]$$

subject to the solution of the field analysis problem; in Equation (2) Ω is the feasible region *i.e.*, the set of admissible positions for antenna 1 on the chest surface (Figure 5). Apparently, a min-max problem is originated: given a feasible solution *x*, the field analysis is performed for a discrete set of frequencies *f_k_*, *k* = 1, *n_f_* (where *n_f_* is equal to 227 for given FFT parameter of FDTD method) within the bandwidth *B* assuming the six-cylinder human body model. Subsequently, *S*_21_ and *S*_31_ scattering parameters are computed for each frequency *f_k_* in the set. Eventually, the objective function value is the highest between *S*_21_ and *S*_31_ values within the bandwidth *B*.

A numerical approximation to the solution of Equation (2) is found by means of the optimization algorithm presented in the following paragraph.

### 2.3. Optimization Algorithm

Over the last decade, evolutionary algorithms (EAs) have proven to be successful in solving optimization problems in the area of computational electromagnetism, see e.g., [22,23,24,25,26]. In fact, EAs do not require the objective function gradient to identify the search direction, and are global-optimum oriented in the case of non-convex objectives. Moreover, they are able to find a solution in a short time when the optimization problem is characterized by several constraints or exhibits discontinuities, while classical methods based on gradient information cannot be applied. Often, EAs are inspired by the models of natural phenomena: For instance, “island” paradigms mimic the phenomenon of populations evolving without exchange with the external environment [27]. In turn, the biogeography-based optimization (BBO) algorithm [28], which is inspired by the geographical distribution of species in an ecosystem, has been applied in the antenna design [29,30]. Particle swarm optimization, which imitates the behavior of a swarm of bees during their food-searching activity, was introduced in the antenna community [31] and stimulated numerous contributions, both under single-objective and under multi-objective context [32]. More recently, wind-driven optimization, inspired by the wind motion, which takes place in the high atmosphere to compensate the pressure imbalance, has been proposed and applied in the antenna array design [33]. Numerous techniques have been considered to reduce optimization time, such as more cost-effective electromagnetic codes (e.g., if applicable, the boundary element method instead of the finite difference time domain method), simplified simulation models (e.g., homogenous body phantoms instead of multi-tissue), massively parallel computing (e.g., using graphics card) or surrogate modelling which may require fewer evaluations of the objective function [34,35]. The approach taken in this paper, which adopts a simplified, homogenous model of the human body, turned out to sufficiently reduce the optimization time combined with an EA EStra [26] lowest order (*i.e.*, a single parent generates a single offspring) algorithm. With the direct approach involving Remcom XFdtd and a heterogeneous human body model the computation time turned out to be excessive, considering a great number of design variants taken into account.

In this paper EStra [27] was used as an evolutionary algorithm of the lowest order (*i.e.*, a single parent generates a single offspring).

In EStra each out of *n_v_* design variables is represented by two pieces of information, *i.e.*, mean value *m*, and standard deviation *d*. The search in the *n_v_*-dimensional design space begins in a region of radius *d*_0_ (initial standard deviation) centered at the starting point *m*_0_ (initial mean value); *m_0_* is externally provided, while *d*_0_ is internally calculated based on the bounds boxing the variation of the design variables. After initialization, the optimization loop is based on three major steps as follows:

- Setting **m** = **m**_0_ and **d** = **d**_0_ at the initial iteration, the generation of the design vector **x** = **m** + d**u** takes place, resorting to a stochastic sample u∈(0,1); generally, *u* is a normally distributed sample. Vector **x** is accepted if it fulfils bounds and constraints; otherwise, a new design vector is generated until the corresponding design point falls inside the feasible region.
- The associated objective function f(**x**) is then evaluated, and the test f(**x**) < f(**m**) is performed; if the test is successful, **m** is replaced by **x** (*mutation* operator), otherwise **m** is conserved.
- The last step rules the size of the search region that will be used for the successive iteration: when a design point that is better than the current one is found, the radius of the search region is increased around the new point to search for further improvements; if no improvement is found, the radius of the search region is gradually decreased, up to convergence.

In contrast to gradient-based methods of numerical optimization, EStra is suitable for non-convex, non-smooth and discontinuous functions; in particular, it is able to approximate the global minimum of the objective function, regardless of the starting point. The implementation of lowest-order EStra originated a cost-effective algorithm of numerical optimization, with respect to a multi-member implementation, where at each iteration a set of parent individuals recombines to generate a set of offspring individuals; this feature is particularly important when the solution of the direct problem relies on a time-consuming field analysis.

In fact, successful applications of EStra to the design of low-frequency electromagnetic setups can be found in [26], while its combination with a FDTD solver for body-worn wireless sensor network optimization is an original concept presented in this paper. The subject is an emerging one. In fact, in [36] a flow optimization model for designing WBAN is provided; attention is focused on network installation cost and energy consumption of antennas, which are both design criteria to minimize. In [9] a robust optimization model and a new algorithm for designing WBAN in terms of topology and routing, taking into account data generation uncertainty, is presented. Also nature-mimicking algorithms are successfully exploited in WBAN design: for instance, [7] presents a fast evolutionary algorithm inspired by an ant colony; the case study is the optimal design of the body sensor network defined in [8].

As far as the implementation of the optimization procedure proposed in this paper is concerned, the following remarks can be put forward:

The XFdtd package provides users with a scripting language based on ECMAScript, in which one can prepare an antenna geometry model, as well as meshing and simulation procedures.

**Figure 6:**
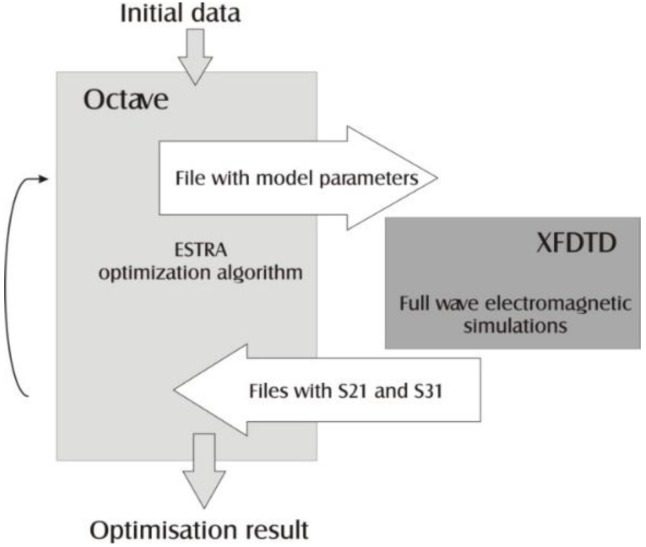
Optimization algorithm flow chart.

In contrast, EStra optimization algorithm was originally coded in Mathworks Matlab and for further work Octave [37] environment was applied because it is freeware and compatible with original Matlab scripts. The Octave and XFdtd scripts are combined and operate in turns to generate a new antenna position and calculate the objective function. Synchronization and data exchange between XFdtd and Octave have been solved by file exchange (Figure 6).

The position of the antenna towards the chest was controlled using a cylindrical coordinate system: *Z_p_*—Antenna vertical position, ϕ*_p_*—Antenna azimuthal position and *d*—Antenna distance to the body surface, presented in Figure 5. Due to the utilization of the simplified cylindrical body model it was easy to use three design variables for creating the antenna location constraints region which was equidistant to the cylindrical body surface.

The numerical model of the network under optimization consists of a human body model (1.8 m tall) and the dipole antennas of 1 mm diameter and the length equal to 53 mm for the 2.4 GHz case and 20 mm for the 5.8 GHz case. In order to decrease the computation time, we chose an adaptive meshing in XFdtd. For the simulations in 2.4 GHz band the voxel within the body model has an edge length equal to 10 mm, and 5 mm for 5.8 GHz. In the regions where antennas were located the voxel size was reduced to 0.5 mm. The model for 2.4 GHz consists of 8,276,136 voxels. With a computer equipped with two Nvidia Tesla C2075 processors the simulation time was ca 30 s. In the case of simulations for 5.8 GHz band, 13,306,865 voxels were used and the simulation time was ca 60 s.

As already clarified, we focus on the effect of the human body impact on the path loss assuming free-space environment in which the person is located. This simplification corresponds to a scenario for outdoor locations where multipath propagation is highly reduced. To simulate this effect an absorbing boundary condition was applied for simulations in Xfdtd.

## 3. Simulations and Results

In the optimization process the chest antenna location constraints were defined as a region by the three following ranges of design variable values: Rotation angle 0.2 rad < ϕ*_p_* < 1.55 rad, antenna vertical position 0 m < *z* < 0.6 m and antenna distance to the body 0 < *d* < 0.01 m. These values follow from the application of our research to the firefighters system under development, in which the defined region corresponds to the possible predetermined localization of the jacket pocket for the node. Due to the presence of other firefighter equipment, it was only possible to place the pocket for the chest node in the left-front part of the jacket (two other sensors have a fixed location on the right arm and the left side of the head). We assumed that the distance between the antenna and the body could be adjusted slightly by placing the pocket on the inner or the outer side of the jacket or by using styrofoam spacers. We also assumed that the optimization process will start from the location of the chest antenna defined by the ϕ*_p_* = 1 rad *Z_p_* = 0.4 m and *d* = 0.001 m (antenna azimuthal position or rotation angle, antenna vertical position, antenna distance to the body surface according to Figure 5). It is the location of an already existing jacket pocket, which we intended to use for the chest module.

During the first stage of the optimization experiment a sub-problem of Equation (2) was considered, defined by Equation (3), where we are considering only two antennas operating in the ISM 2.4 GHz band: the head antenna with a fixed position and the chest antenna with a variable position.

(3) $$\mathrm{Find}\;\underset{x\in \Omega }{\inf}\;\underset{f\in B}{\sup}\;S_{21}\left(x,f\right)\;,\;x=\left(z_{p},\varphi_{p}\right)\;,\;\Omega \subset {ℜ}^{3}\;,\;B=\left[f_{L},f_{H}\right]$$

with *f_L_* = 2.4 GHz and *f_H_* = 2.5 GHz.

**Figure 7:**
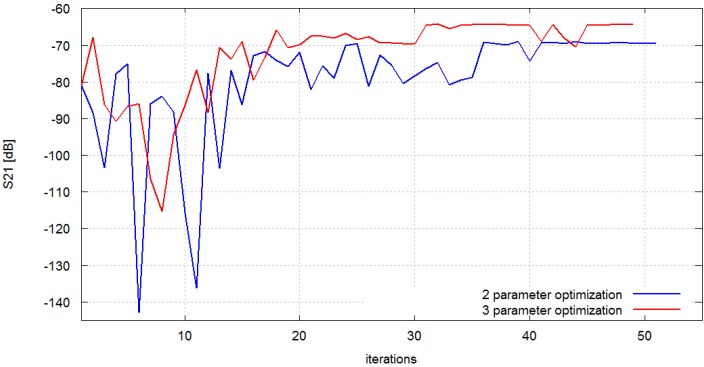
Optimization process for the two-antenna scenario; the objective function is *S*_21_.

Therefore in Equation (3) only S_21_ is present. The objective function (to be maximized) was chosen as the minimum value of S_21_ in the 2.4–2.5 GHz frequency band. Consequently, a two-variable optimization was considered first. The third design variable—Antenna distance—Was fixed at *d* = 0.001 m. The results obtained after 51 iterations are presented in Figure 7 (blue line). The initial value of *S*_21_ objective function was −81 dB and the final was −69 dB. We may observe that the radio link quality has improved because the signal received in the optimized case has 12 dB (−81 dB *vs.* −69 dB) greater amplitude.

Subsequently, the full problem defined by Equation (2) was considered, *i.e.*, optimization experiments with two antennas operating at 2.4 GHz was repeated, but this time all three variables of the chest antenna position were identified (antenna rotation angle, antenna vertical position, antenna distance to the surface of the body). The results obtained after 49 iterations are presented in Figure 7 (red line). The initial value of *S*_21_ objective function was −81 dB, and the final was −64 dB. This means that also in this case the link quality has improved because the signal received in the optimized setup has 17 dB greater amplitude. It can be concluded that the three-variable optimization may provide a greater improvement of the objective function. This result may seem trivial considering that a better solution should exist in the three-parameter optimization space compared to the two-parameter optimization space. However, the problem is that this solution may not be found a particular optimization algorithm. The improvement achieved in our experiments suggests that EStra algorithm is suitable for the class of problems under consideration.

**Figure 8:**
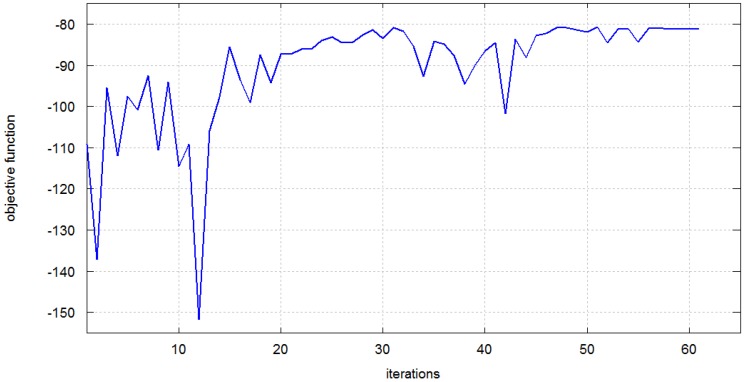
Optimization process with the three-antenna scenario; the objective function is the minimum of *S*_21_ and *S*_31_.

We used the three-variable approach to optimize the three-antenna scenario operating at 2.4 GHz. In this case the objective function was defined as the minimum value from among two parameters: *S*_21_ and *S*_31_ in the frequency range: 2.4–2.5 GHz. For the same starting point as previously, the initial value of objective function was −109 dB. After 61 iterations it was improved to −80 dB, which is a gain of 29 dB in the radio link budget. The improvement of the objective function in the optimization process *vs.* iteration number is presented in Figure 8.

**Figure 9:**
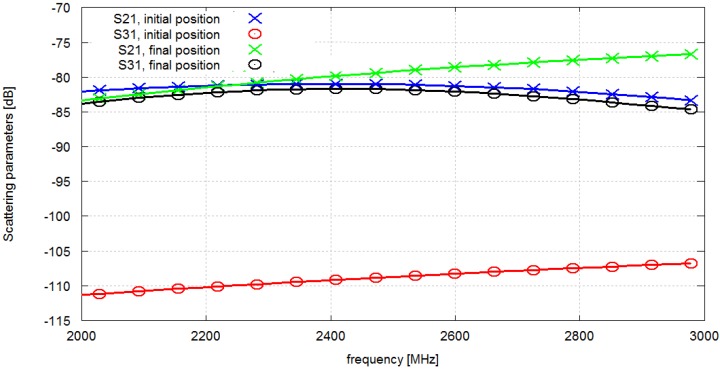
Scattering matrix parameter values *vs.* frequency before and after optimization for 2.4 GHz frequency band.

In Figure 9 we present the scattering matrix parameter values (*S*_21_ and *S*_31_) before and after optimization for 2.4 GHz. The improvement of *S*_31_ is visible while *S*_21_ changed only slightly.

**Figure 10:**
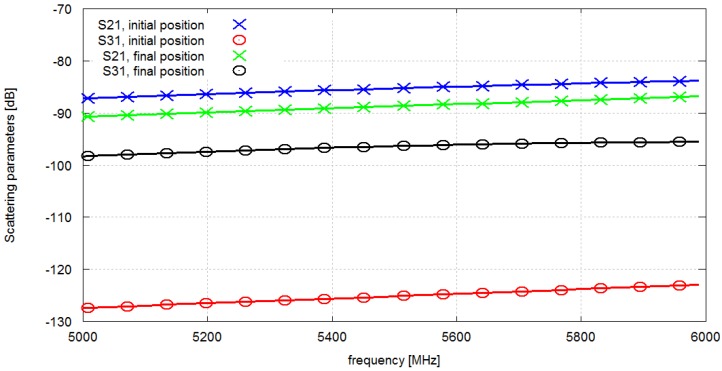
Scattering matrix parameter values *vs.* frequency before and after optimization for 5.8 GHz frequency band.

The good performance of the optimization procedure that we observed at 2.4 GHz was then verified in the 5.8 GHz ISM band. For this purpose we used shorter dipoles (20 mm long) which were tuned for the frequency equal to 5.8 GHz. The optimization starting point and the objective function definition were the same as for 2.4 GHz. The results were obtained after 67 iterations. The initial value of the objective function (the lowest value of *S*_21_ and *S*_31_ within the frequency range from 5.7 GHZ to 5.9 GHz) was −124 dB and it improved after 67 iterations to −96 dB. Figure 10 presents the scattering matrix parameter values before and after optimization. The results of all the described optimization experiments are gathered in Table 1.

**Figure 11:**
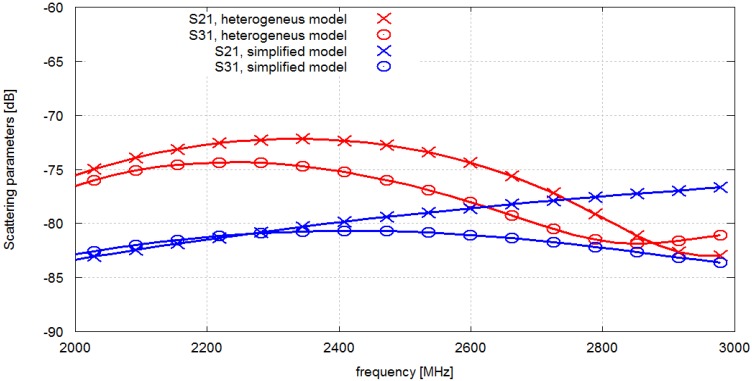
Comparison of results obtained with simplified model and heterogeneous model.

We have assessed the validity of our simplified human body model using a much more detailed numerical anthropomorphic, heterogeneous body model (NMR Hershey) which is available in Remcom Xfdtd. We selected the model version comprising 5 mm voxels. The final antenna position obtained in the optimization process was applied to the model of the sensor network but this time with the heterogeneous numerical phantom. The simulations were performed at 2.4 GHz with three antennas/nodes. The results are presented in Figure 11. A good agreement between the values obtained with the simplified model and the heterogeneous model can be observed, which advocates for the usage of the simplified one.

## 4. Conclusions

In this paper we present original results of our investigation of an evolutionary optimization of a simple body-worn wireless sensor network comprising three on-body nodes. This case was motivated by the practical need of improving performance of a WBAN designed to monitor firefighters’ vital signs and environmental conditions. A number of design assumptions and constraints were imposed by our application, for example the fixed position of the head and arm nodes predetermined by the types of sensors they use. One degree of freedom available to us was the placement of the chest node in the front-left part of the jacket. In this paper we show that even with such strong design constraints it is possible to considerably decrease the path loss between nodes using an automated optimization procedure based on evolutionary computing (EStra) combined with a computational electromagnetics code (XFdtd) to evaluate the value of the objective function. Depending on the starting point, the improvement will differ but in our experiments it reached about 20 dB. This improvement would result in reduced power consumption of the WBAN node transmitters.

The three-variable optimization that allowed the change of both the antenna position and the antenna distance to the human body provided better results than the two-variable optimization with a fixed distance. However, this result may be difficult to apply in practice due to the difficulty of controlling the distance e.g., by adding dielectric spacers into the jacket.

We show that the proposed methodology of sensor network optimization provides satisfactory results both in the 2.4 GHz band and in the 5.8 GHz band. Since the simplified human body model was positively verified for a broad range of frequencies [20], probably the same optimization routine could be successfully applied in other frequency bands.

We also show that the path loss values obtained at 2.4 GHz with the simplified homogeneous model are comparable to the results achieved with a much more detailed heterogeneous anthropomorphic multi-tissue model. In our study, one advantage of using the simplified cylindrical model is the ease of defining and controlling the antenna displacement with the rotation angle and height only, while keeping a constant antenna distance to the body. Another advantage is reduced computation time resulting from sparser gridding because the complexity of inner organs does not have to be represented. Our approach to the optimization problem is computationally effective. A single iteration lasted less than one minute and the overall simulation time was well below one hour.

Further investigation is planned for ultra wideband WBAN optimization.

## Figures and Tables

**Table 1:** Optimization with the evolution strategy algorithm.

	Optimization Scenario			
	2 Antennas, 2.4 GHz Fixed Distance	2 Antennas, 2.4 GHz Variable Distance	3 Antennas, 2.4 GHz Variable Distance	3 Antennas, 5 GHz Variable Distance
**Final Value of Angle [rad]**	0.63	0.5	0.22	0.35
**Final Value of Vertical Coordinate [m]**	0.567	0.379	0.18	0.335
**Final Value of Antenna Distance [m]**	0.001	0.007	0.007	0.0045
**Initial Value of Objective Function [dB]**	−81	−81	−109	−124
**Final Value of Objective Function [dB]**	−69	−64	−80	−96
**Improvement of Objective Function [dB]**	12	17	29	28
